# Large Language Models for Traumatic Dental Injuries Across Web‐Based and Mobile‐Based Interfaces: Assessing Accuracy, Quality, and Temporal Consistency

**DOI:** 10.1002/cre2.70416

**Published:** 2026-07-19

**Authors:** Ezgi Can Cekic, Mertkan Kumru, Burcu Yilmaz, Berk Celikkol, Oğuz Tavsan

**Affiliations:** ^1^ Department of Endodontics, Faculty of Dentistry Usak University Usak Türkiye

**Keywords:** artificial intelligence, dentistry, endodontics, information reliability, natural language processing, traumatic dental injuries

## Abstract

**Objectives:**

Traumatic dental injuries (TDIs) are frequent in clinical practice and require rapid, guideline‐based decisions, yet accessing accurate and reliable information may be challenging. Large language models (LLMs) such as ChatGPT, Gemini, DeepSeek, and Qwen are increasingly used as quick online information tools; however, evidence regarding their accuracy, consistency, and the influence of different user interfaces is limited. This study aimed to evaluate the performance of several LLMs in answering TDI‐related questions through both web‐based interfaces and mobile phone applications.

**Material and Methods:**

Twenty questions were prepared according to the 2020 International Association of Dental Traumatology (IADT) guidelines, including 10 open‐ended and 10 yes–no items. Four LLMs (ChatGPT‐4o, DeepSeek‐V3, Gemini 2.0 Flash, Qwen2.5‐Max) were queried simultaneously via web and mobile interfaces over five consecutive days, generating 800 responses. Open‐ended answers were assessed using the Global Quality Score (GQS) and modified DISCERN (mDISCERN), while yes–no responses were compared with a predetermined answer key. Statistical analyses were performed using IBM SPSS v23.0, with significance set at *p* < 0.05.

**Results:**

Qwen2.5‐Max demonstrated comparatively higher GQS and mDISCERN scores across both interfaces. Accuracy for yes–no questions ranged from 86% to 91% without significant differences among models. Interface comparisons showed that ChatGPT‐4o generated comparatively higher‐quality responses on the web, whereas Qwen2.5‐Max performed better on mobile. Over the 5‐day period, Qwen2.5‐Max showed relatively higher temporal consistency, while DeepSeek‐V3 exhibited notable day‐to‐day variation.

**Conclusions:**

LLMs may serve as useful supplementary tools for providing guideline‐based information on TDIs, especially for straightforward, closed‐ended clinical questions. However, their performance varies by model, interface, and question type. Qwen2.5‐Max demonstrated comparatively higher performance across several evaluated measures. Despite these results, LLM‐generated information should be interpreted cautiously and verified by dental professionals before being used in clinical decision‐making.

## 1 Introduction

Traumatic dental injuries (TDIs) represent a significant public health concern, particularly among children and adolescents, affecting more than one billion individuals worldwide (Petti et al. 2018). Various types of TDIs, such as fractures, luxations, and avulsions, may lead to functional impairments, aesthetic deficiencies, and psychological stress. Achieving optimal clinical outcomes requires timely intervention based on evidence‐based management protocols (Khan and Jindal 2023). The 2020 guidelines published by the International Association of Dental Traumatology (IADT) provide such recommendations to improve clinical outcomes (Levin et al. 2020; Bourguignon et al. 2020; Fouad et al. 2020; Day et al. 2020). However, rapid access to reliable information remains challenging for both clinicians and patients, especially in emergency situations such as avulsion injuries that require immediate intervention (Mustuloğlu and Deniz 2025; Çege et al. 2025).

AI‐based chatbots, also known as large language models (LLMs), have emerged as accessible tools for delivering health information and supporting clinical decision‐making (Sallam et al. 2025; Qiang et al. 2025). In time‐sensitive situations such as TDIs, identifying the appropriate management strategy through comprehensive guideline consultation may not always be practical, particularly for patients and less experienced clinicians. In this context, LLMs can provide rapid, structured, and user‐friendly responses to clinical queries, making them a potentially valuable alternative source of (Sallam et al. 2025; Qiang et al. 2025). However, given the potential variability in the accuracy and reliability of LLM‐generated responses, especially in critical clinical scenarios, systematic evaluation of their reliability becomes essential. Although LLMs have been increasingly studied in healthcare and dentistry, research focusing specifically on their use in TDI‐related scenarios remains limited (Mustuloğlu and Deniz 2025; Çege et al. 2025; Guven et al. 2025).

Previous studies have explored LLM performance in terms of usability (Ayorinde et al. 2024), perceived reliability (Zieglmeier and Lehene 2021), and the influence of presentation format on clinical decision‐making (Tang et al. 2023). However, despite the widespread use of both web‐based and mobile‐based platforms, there is no study directly comparing the performance of the same LLM across these interfaces. This represents a critical gap, as differences in interface design and output presentation may influence the accuracy, reliability, and consistency of information provided in clinical contexts. Therefore, this study aims to evaluate the performance of LLMs in the context of TDIs, with a particular focus on differences between web‐based and mobile‐based interfaces. The analysis includes response accuracy, general quality (GQS), information reliability (mDISCERN), and temporal consistency across repeated queries. In addition, the accuracy of responses to yes–no questions was assessed separately.

In this context, the study seeks to address the following key questions:

- How do LLMs perform in terms of accuracy, general quality (GQS), and information reliability (mDISCERN) in TDI‐related queries?
- What is the level of temporal consistency in model responses across different days?
- Does the interface (web‐based vs. mobile‐based) influence the accuracy and reliability of responses?

The findings are expected to contribute to the development of AI‐assisted clinical decision support systems in dental trauma management and to support improved access to reliable information for both clinicians and patients.

## 2 Materials and Methods

This observational study did not involve any clinical interventions or the use of identifiable patient data; therefore, formal ethical approval was not required. The research process was conducted in accordance with the principles of the Declaration of Helsinki.

The question set used in this study was developed based on the 2020 guidelines on traumatic dental injuries published by the International Association of Dental Traumatology (IADT) (Levin et al. 2020; Bourguignon et al. 2020; Fouad et al. 2020; Day et al. 2020). Of the total 20 questions, 10 were open‐ended, and 10 were in a yes–no format. The questions were prepared by two endodontists with at least 5 years of clinical experience and were reviewed by a third independent endodontist to ensure adequate representation of the main topics of the guideline, clinical relevance, and clarity of wording. After the necessary revisions, the final version of the questions was established. The number of questions was determined to ensure coverage of major TDI scenarios rather than statistical sampling, and the 5‐day period was selected to evaluate short‐term response stability under controlled conditions.

The data collection process began on May 5, 2025, and was conducted at 9:00 a.m. on five consecutive days. Two separate accounts were created for each model: one operated through the web‐based interface and the other through the mobile‐based interface. The questions were presented to both interfaces simultaneously and in the same order. After each response, the conversation history was cleared to ensure that all questions were answered independently. This approach aimed to allow for an objective comparison of performance between interfaces and to eliminate the risk of previous responses influencing subsequent model outputs.

Four different large language models (LLMs) were evaluated in this study: ChatGPT‐4o (OpenAI, San Francisco, USA), DeepSeek‐V3 (DeepSeek AI, China), Gemini 2.0 Flash (Google DeepMind, London, UK), and Qwen2.5‐Max (Alibaba Cloud, Hangzhou, China). These models were selected to include both widely studied systems (e.g., ChatGPT‐4o, Gemini) and relatively newer, less extensively researched systems (e.g., DeepSeek‐V3, Qwen2.5‐Max). This approach enabled the comparison of LLMs with varying levels of maturity and adoption within the same framework in TDI scenarios (Sallam et al. 2025; Guven et al. 2025).

The responses obtained from the chatbots were evaluated by two independent endodontists who did not participate in the data collection process, in order to minimize researcher bias during the scoring process. The evaluators were trained in advance on the scoring criteria and conducted a pilot pre‐test on 20 randomly selected questions. This pre‐test was performed to ensure inter‐rater agreement. During the calibration phase, inter‐rater reliability was calculated using the intraclass correlation coefficient (ICC), based on a two‐way mixed‐effects model with absolute agreement definition (average measures), yielding values of 0.89 for GQS and 0.91 for mDISCERN, indicating a high level of agreement.

The responses were evaluated across three dimensions:

- mDISCERN score (0–5): Responses were scored based on clarity, use of references, neutrality, mention of alternative treatment options, and evidence‐based content (Table 1) (Kilinc and Sayar 2019).
- General Quality Score (GQS, 1–5): The overall information quality, educational value, and clinical applicability of the responses were subjectively assessed (Table 1) (Kilinc and Sayar 2019).
- Yes–no questions: The correctness of responses was evaluated based on a predefined answer key derived directly from the 2020 IADT guidelines, which served as the ground truth reference standard, and the percentage of correct answers was calculated (Levin et al. 2020; Bourguignon et al. 2020; Fouad et al. 2020; Day et al. 2020).

**Table 1:** Assessment tools for evaluating the reliability (mDISCERN) and general quality (GQS) of LLM responses.

**Modified DISCERN (If the answer is “yes,” one point is awarded for each question)**	
**1.** Is the response presented clearly, concisely, and understandably?	
**2.** Are reliable references or guideline citations provided to support the response?	
**3.** Is the information presented in a balanced and unbiased manner (including mention of alternative treatment options)?	
**4.** Are additional information sources or further readings suggested?	
**5.** Does the response address areas of uncertainty?	

**GQS**	**Description**
**1**	Poor quality, poor flow, most information missing, not useful for education.
**2**	Generally, poor quality and flow, of limited use to patients because only some information is present, but many important topics are missing.
**3**	Moderate quality, suboptimal flow, somewhat useful for patients as some important information is adequately discussed, but others are poorly discussed.
**4**	Good quality, generally good flow, useful to patients because most relevant information is covered, but some topics are not covered.
**5**	Excellent quality and flow; complete, educational, and highly applicable in clinical practice.

### 2.1 Statistical Analysis

All statistical analyses were performed using IBM SPSS Statistics v23.0 (IBM Corp., Armonk, NY, USA). Continuous variables were expressed as mean ± standard deviation (SD) or mean ± standard error (SE), while categorical variables were presented as frequency (n) and percentage (%). A two‐tailed *p*‐value of < 0.05 was considered statistically significant, and the results were reported with 95% confidence intervals (CIs). To account for the non‐independence of repeated responses across five consecutive days, scores obtained for each question were aggregated by calculating the mean values for each model and interface prior to comparative statistical analyses. This approach ensured that the assumptions of independence required for parametric tests were not violated.

For the analysis of open‐ended questions, one‐way analysis of variance (ANOVA) was used to evaluate the differences in mean scores between the models. In cases where statistically significant differences were detected, Tukey's Honest Significant Difference (HSD) post‐hoc test was applied to determine the source of the differences.

For the interface comparison, the scores for the web‐based and mobile‐based interfaces were analyzed using a paired‐samples *t*‐test. The results of the analysis were reported with the mean difference, *t*‐statistic, and *p*‐value.

For the temporal consistency analysis, the intraclass correlation coefficient (ICC) was calculated using a two‐way random‐effects model with absolute agreement definition to assess the consistency of GQS and mDISCERN scores across days. The results were presented with 95% confidence intervals and variance components (day, question, and residual).

For the analysis of yes–no questions, Pearson's Chi‐square (*χ* ^2^) test was applied for the comparison of categorical data, and effect size was calculated using Cramér's V or Phi (*Φ*) coefficient depending on the number of variable categories. For pairwise comparisons between interfaces, Yates' continuity‐corrected Chi‐square test was used, while Bonferroni‐corrected z‐tests were applied for multiple comparisons. The temporal consistency of these questions across days was assessed using Fleiss' *κ* coefficient and interpreted according to the Landis and Koch (1977) classification (*κ* < 0.00: poor, 0.00–0.20: slight, 0.21–0.40: fair, 0.41–0.60: moderate, 0.61–0.80: substantial, and 0.81–1.00: almost perfect).

## 3 Results

In this study, the performances of four large language models (LLMs) in the field of TDIs were evaluated over five consecutive days, and a total of 800 responses (4 models × 2 interfaces × 20 questions × 5 days) were collected. For comparative analyses, responses were aggregated at the question level across days. The data obtained were examined in terms of response quality and information reliability for open‐ended questions, accuracy rates for yes–no questions, and temporal consistency of responses across days. In addition, performance differences between web and mobile‐based interfaces were compared.

### 3.1 Response Quality and Information Reliability for Open‐Ended Questions (GQS and mDISCERN)

Mean scores reported reflect aggregated values across five consecutive days. The mean (±SE) GQS and mDISCERN scores of the models' responses to open‐ended questions are presented in Table 2. One‐way ANOVA analyses revealed statistically significant differences among the models for both GQS and mDISCERN scores (*p* < 0.001). Tukey HSD post‐hoc tests showed that Qwen2.5‐Max achieved significantly higher scores than the other models in both the web‐based (GQS: 4.08 ± 0.07) and mobile‐based interfaces (GQS: 4.32 ± 0.07) (*p* < 0.05). Similarly, Qwen2.5‐Max exhibited statistically significantly higher mean mDISCERN scores across both interfaces (web: 3.13 ± 0.06; mobile: 3.34 ± 0.06) (*p* < 0.05). DeepSeek‐V3 produced lower scores than Qwen2.5‐Max but higher scores than Gemini 2.0 Flash and ChatGPT‐4o for both measures (*p* < 0.05). Gemini 2.0 Flash had mean scores between those of DeepSeek‐V3 and ChatGPT‐4o, whereas ChatGPT‐4o reached the lowest GQS (3.23 ± 0.10) and mDISCERN (2.42 ± 0.07) values in the mobile‐based interface.

**Table 2:** Mean (±SE) GQS and mDISCERN scores of large language models' responses to open‐ended questions across web‐based and mobile‐based user interfaces.

Platform	Metric	ChatGPT‐4o (mean ± SE)	DeepSeek‐V3 (mean ± SE)	Gemini 2.0 Flash (mean ± SE)	Qwen2.5‐Max (mean ± SE)	*F* statistic (one‐way ANOVA)	*p* value
Web	GQS	3.65 (0.10)^c^	3.75 (0.10)^b^	3.23 (0.10)^c^	4.08 (0.07)^a^	15.632	< 0.001
	mDISCERN	2.82 (0.07)^b^	2.99 (0.07)^b^	2.71 (0.07)^c^	3.13 (0.06)^a^	9.876	< 0.001
App	GQS	3.23 (0.10)^c^	3.61 (0.10)^b^	3.42 (0.10)^b^	4.32 (0.07)^a^	20.145	< 0.001
	mDISCERN	2.42 (0.07)^d^	2.88 (0.07)^b^	2.62 (0.07)^c^	3.34 (0.06)^a^	18.765	< 0.001

*Note:* Values represent mean ± standard error (SE). *F*‐statistic values were obtained using one‐way analysis of variance (ANOVA). Post‐hoc comparisons were performed with Tukey's HSD test; means with the same superscript letter within the same platform (web or app) are not significantly different at *p* < 0.05. Values represent aggregated mean scores across five consecutive days.

Abbreviations: App, mobile‐based user interfaces; GQS, Global Quality Score; mDISCERN, modified DISCERN; *p*, *p*‐value; SE, standard error; Web, web‐based user interfaces.

### 3.2 Accuracy Rates for Yes–No Questions

**Figure 1:**
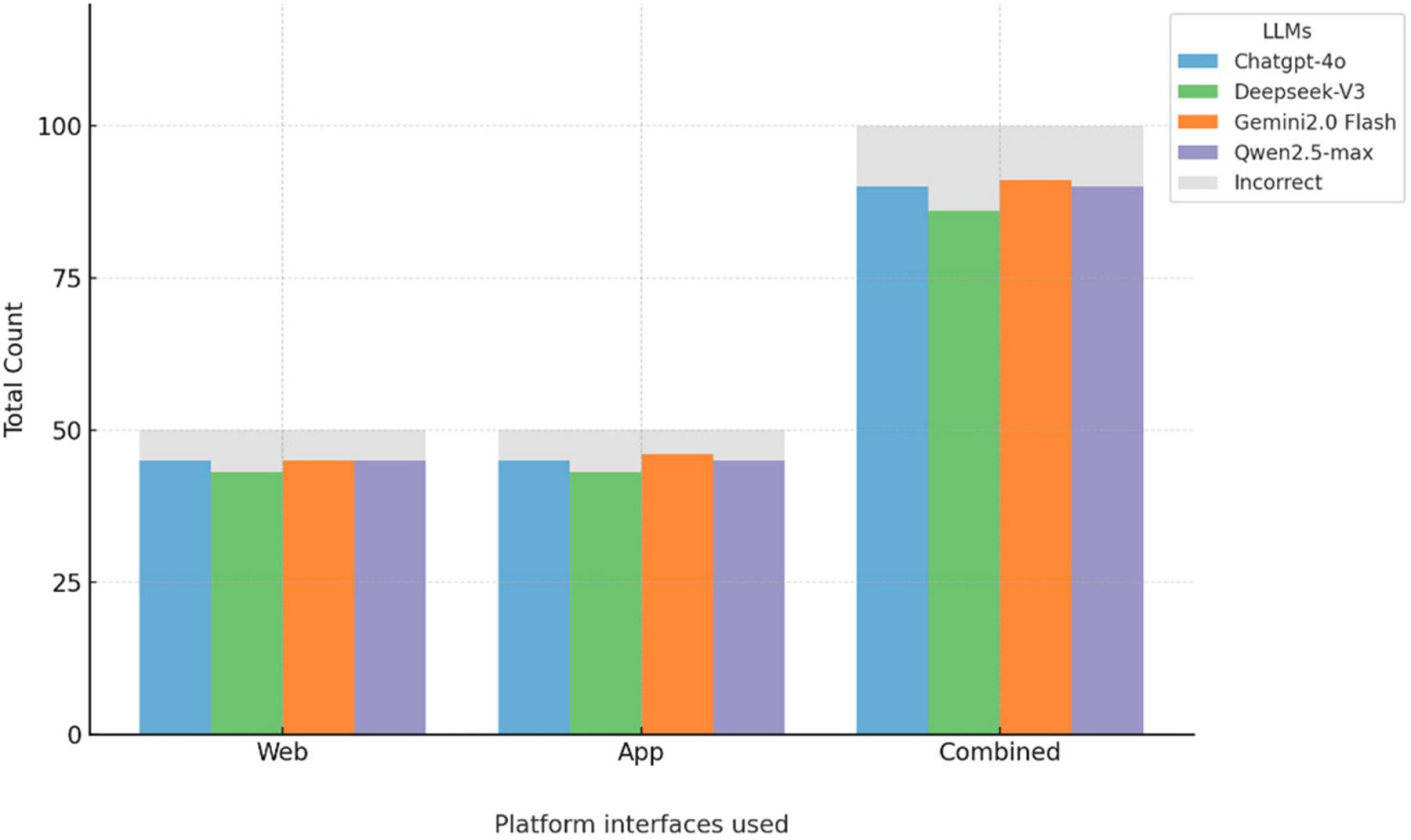
Distribution of large language models' (LLMs) responses to correct and incorrect question types across web‐based, mobile‐based, and combined user interfaces. App: mobile‐based interface; LLMs: large language models; Web: web‐based interface.

The yes–no format questions were evaluated using a pre‐prepared standard answer key. Pearson's Chi‐square test revealed no statistically significant difference among the models (web: *χ* ^2^[3] = 0.61, *p* = 0.893; mobile: *χ* ^2^[3] = 1.01, *p* = 0.799). The accuracy rates of all models were high, ranging between 86% and 91%. These results indicate that there was no significant performance difference among the models for the yes–no question format (Figure 1).

### 3.3 Comparison of Web‐Based and Mobile‐Based Interface Performance

#### 3.3.1 Open‐Ended Questions

Paired‐samples *t*‐test analyses showed that ChatGPT‐4o produced significantly higher GQS (difference=0.42, *p* < 0.001) and mDISCERN (difference=0.40, *p* = 0.003) scores on the web‐based interface compared to the mobile‐based interface. Conversely, Qwen2.5‐Max generated significantly higher scores on the mobile‐based interface than on the web‐based interface (GQS: difference = −0.24, *p* = 0.018; mDISCERN: difference = −0.21, *p* = 0.027). No significant differences were observed between interfaces for DeepSeek‐V3 and Gemini 2.0 Flash (all *p* > 0.05). These findings indicate that model performance on open‐ended questions may vary depending on the interface used (Table 3).

**Table 3:** Comparison of large language models' performance on open‐ended questions across web‐based and mobile‐based user interfaces.

Bot	Metric	Web (mean ± SE)	App (mean ± SE)	Difference (web–app)	*t*‐test statistic	*p* value
ChatGPT‐4o	GQS	3.65 ± 0.10	3.23 ± 0.10	0.42	4.584	< 0.001*
	mDISCERN	2.82 ± 0.07	2.42 ± 0.07	0.40	2.958	0.003*
DeepSeek‐V3	GQS	3.75 ± 0.10	3.61 ± 0.10	0.14	1.035	0.301
	mDISCERN	2.99 ± 0.07	2.88 ± 0.07	0.11	1.627	0.104
Gemini 2.0 Flash	GQS	3.23 ± 0.10	3.42 ± 0.10	–0.19	–1.331	0.184
	mDISCERN	2.71 ± 0.07	2.62 ± 0.07	0.09	0.444	0.658
Qwen2.5‐Max	GQS	4.08 ± 0.07	4.32 ± 0.07	–0.24	–2.366	0.018*
	mDISCERN	3.13 ± 0.06	3.34 ± 0.06	–0.21	–2.218	0.027*

*Note:* Paired samples *t*‐test; values are presented as mean (±SE); *t*‐test statistic (*t*) and *p*‐values are reported. *p* < 0.05 was considered statistically significant.

Abbreviations: App, mobile‐based interface; GQS, Global Quality Score; mDISCERN, modified DISCERN; *p*, *p*‐value; SE, standard error; Web, web‐based interface.

* Statistically significant at *p* < 0.05.

#### 3.3.2 Yes–No Questions

The accuracy rates for yes–no questions were found to be similar between the web‐based and mobile‐based interfaces, with no statistically significant differences observed among the models (all *p* > 0.05). The graphical distribution demonstrates that all models exhibited high and closely aligned accuracy rates across both interfaces (Figure 1).

### 3.4 Temporal Consistency of Responses

#### 3.4.1 Open‐Ended Questions

**Figure 2:**
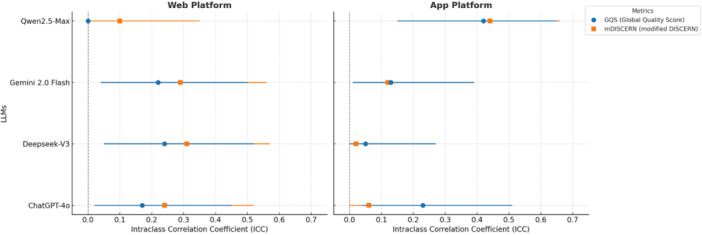
Temporal (inter‐day) consistency of large language model (LLM) responses across web and app platforms, assessed using Intraclass Correlation Coefficient (ICC) with 95% confidence intervals. Blue circles represent GQS (Global Quality Score) and orange squares represent mDISCERN (modified DISCERN instrument) values. Points indicate ICC estimates, and horizontal lines denote 95% confidence intervals. The vertical dashed line at 0 represents the threshold of no agreement. App: mobile‐based interface; GQS: Global Quality Score; ICC: intraclass correlation coefficient; LLMs: large language models; mDISCERN: modified DISCERN; Web: web‐based interface.

The consistency of responses to repeated open‐ended questions over 5 days was evaluated using the intraclass correlation coefficient (ICC), with detailed results presented in Table 4. The graphical distribution of ICC values is shown in Figure 2. Qwen2.5‐Max demonstrated the highest consistency on the mobile‐based interface for both GQS (ICC = 0.42, 95% CI: 0.15–0.65) and mDISCERN (ICC = 0.44, 95% CI: 0.16–0.66). DeepSeek‐V3 showed the lowest day‐to‐day consistency for GQS (ICC = 0.05) and did not exhibit statistically significant stability.

**Table 4:** Temporal (inter‐day) consistency of LLM responses to open‐ended questions across web‐based and mobile‐based interfaces over a 5‐day evaluation period.

Platform	Metric	Bot	ICC (95% CI)	Var (day)	Var (question)	Var (residual)
Web	GQS	ChatGPT‐4o	0.17 (0.02–0.45)*	0.098	0.134	0.480
		DeepSeek‐V3	0.24 (0.05–0.52)*	0.165	0.134	0.530
		Gemini 2.0 Flash	0.22 (0.04–0.50)*	0.193	0.134	0.680
		Qwen2.5‐Max	0.00 (0.00–0.00)	0.002	0.134	0.400
	mDISCERN	ChatGPT‐4o	0.24 (0.05–0.52)*	0.095	0.134	0.300
		DeepSeek‐V3	0.31 (0.08–0.57)*	0.082	0.134	0.180
		Gemini 2.0 Flash	0.29 (0.07–0.56)*	0.091	0.134	0.220
		Qwen2.5‐Max	0.10 (0.00–0.35)	0.027	0.134	0.240
App	GQS	ChatGPT‐4o	0.23 (0.04–0.51)*	0.257	0.134	0.850
		DeepSeek‐V3	0.05 (0.00–0.27)	0.069	0.134	1.220
		Gemini 2.0 Flash	0.13 (0.01–0.39)*	0.166	0.134	1.090
		Qwen2.5‐Max	0.42 (0.15–0.65)*	0.243	0.134	0.330
	mDISCERN	ChatGPT‐4o	0.06 (0.00–0.29)	0.028	0.134	0.460
		DeepSeek‐V3	0.02 (0.00–0.20)	0.011	0.134	0.710
		Gemini 2.0 Flash	0.12 (0.01–0.38)	0.070	0.134	0.540
		Qwen2.5‐Max	0.44 (0.16–0.66)*	0.242	0.134	0.310

*Note:* Intraclass correlation coefficients (ICC) are presented with 95% confidence intervals. Variance components (day, question, residual) were calculated using a two‐way random‐effects model.

Abbreviations: App, mobile‐based interface; CI, confidence interval; GQS, Global Quality Score; ICC, intraclass correlation coefficient; mDISCERN, modified DISCERN instrument; Web, web‐based interface.

* It indicates that the lower bound of the confidence interval is above zero, signifying statistically significant reliability of the ICC estimate.

#### 3.4.2 Yes–No Questions

The consistency of yes–no questions was assessed using Fleiss' *κ* coefficient. Qwen2.5‐Max demonstrated perfect consistency across both interfaces (*κ* = 1.0). DeepSeek‐V3 showed substantial consistency on the web‐based interface (*κ* = 0.751) and moderate consistency on the mobile‐based interface (*κ* = 0.419). ChatGPT‐4o exhibited moderate consistency on the web‐based interface (*κ* = 0.556) and perfect consistency on the mobile‐based interface (*κ* = 1.0). When the interfaces were combined, Qwen2.5‐Max maintained perfect consistency, whereas the *κ* value for DeepSeek‐V3 declined to a fair level (*κ* = 0.336).

## 4 Discussion

This study comprehensively evaluated the performance of LLMs in providing information on TDIs, focusing on response accuracy, GQS, mDISCERN, temporal consistency, and performance differences across interfaces of the same model. Overall, the findings suggest that LLM‐generated responses may vary according to both model type and interface, particularly for open‐ended questions. While yes–no questions generally showed similar accuracy across models, greater variability was observed in qualitative measures such as GQS, mDISCERN, and temporal consistency. In addition, certain models demonstrated differences between web‐based and mobile‐based interfaces, suggesting that response characteristics may not be entirely platform‐independent.

One of the most notable findings of our study was that all models showed similar and consistently high accuracy rates in yes–no questions, ranging between 86% and 91%. These findings suggest that LLMs may produce more consistent outputs in closed‐ended question formats. One possible explanation is that closed‐ended formats restrict the response space and reduce ambiguity compared with open‐ended questions.

The literature also supports this observation. Rasool et al. (2024) reported that LLMs achieved higher accuracy in yes–no and single‐choice tasks compared with open‐ended questions in document‐based question–answer settings. Similarly, Künzle and Paris (2024) demonstrated that AI models reached meaningful success levels in standard multiple‐choice examinations in dentistry. Likewise, recent studies evaluating LLM performance in Turkish dental specialty examination questions in oral pathology and head and neck anatomy also reported relatively high performance in multiple‐choice formats, although substantial variability among models was observed depending on the specialty domain and question content (Gokkurt Yilmaz et al. 2025; Yilmaz et al. 2025). Guven et al. also reported that ChatGPT‐3.5 and Gemini achieved high accuracy rates in yes–no questions derived from IADT guidelines. However, the same research group emphasized in a separate study based on open‐ended questions from patient forums that the quality and readability of model responses varied considerably (Guven et al. 2025). These differences may also reflect variations in model architecture, training data, and response‐generation strategies; however, such explanations remain speculative because these systems operate as largely closed‐source platforms.

For open‐ended questions, ChatGPT‐4o, DeepSeek‐V3, and Gemini 2.0 Flash showed moderate performance in terms of GQS and mDISCERN. The main shortcomings of these models were that responses were often superficial, included limited references to guidelines, provided insufficient discussion of alternative treatment options, and failed to clearly address risks and uncertainties. These findings are consistent with previous studies. For example, Kuru et al. (2025) reported that LLMs struggled to deliver reliable information for open‐ended questions, particularly scoring low in reference use and coverage of alternative treatment options. Similarly, Guven et al. (2025) and Johnson et al. (2025) found that the overall information quality of model responses was moderate and highlighted deficiencies in evidence‐based content. In contrast, Qwen2.5‐Max achieved higher scores than the other models in both GQS and mDISCERN (web: 4.08 ± 0.07 and 3.13 ± 0.06; mobile: 4.32 ± 0.07 and 3.34 ± 0.06) and also showed the highest day‐to‐day consistency (mobile ICC = 0.42–0.44). A possible explanation for the relatively higher performance of Qwen2.5‐Max may be that its responses were generally more comprehensive and guideline‐oriented. This finding is also supported by recent studies (Sallam et al. 2025; Zhu, Hu et al. 2025; Zhu, Zhang et al. 2025). Zhu, Hu et al. (2025) reported that Qwen2.5 achieved the highest accuracy among seven LLMs and excelled particularly in complex, multi‐step reasoning tasks. In dentistry, another study demonstrated that Qwen2.5 produced reliable and consistent long‐text outputs with low levels of hallucination (Zhu, Zhang et al. 2025). Moreover, multilingual evaluations showed that Qwen2.5 performed on par with ChatGPT‐4 and even surpassed it in certain scenarios (Sallam et al. 2025). Nevertheless, given the observational nature of the present study and the limited scope of the evaluated questions, these findings should be interpreted cautiously and should not be generalized beyond the specific scenarios assessed.

The variation observed in responses across different days may be related to the intrinsic functioning of LLMs. As highlighted in the literature, LLMs operate in a non‐deterministic manner; thus, even when the same inputs are repeated, variations in word selection driven by probability distributions may produce different outputs (Atil et al. 2024). In addition, prompt sensitivity can affect response reliability with minor changes in wording (Anagnostidis and Bulian 2024), and even input order sensitivity may lead to significant differences in outcomes (Guan et al. 2025). Zhou et al. (2024) further noted that larger and more extensively fine‐tuned models, while generally more successful in complex tasks, may also experience stability loss in certain scenarios. To minimize such variations, we ensured that input content remained identical across days. Nevertheless, the relatively low day‐to‐day consistency observed for some models suggests that the reliability of LLM‐generated information may remain a concern in clinically sensitive contexts such as TDIs. Hackl et al. (2023) reported that GPT‐4 demonstrated high temporal consistency across different time points. However, consistency should not be interpreted as a direct indicator of accuracy or clinical reliability. Indeed, another study in endodontics found that ChatGPT produced consistent responses in yes–no format questions across 10 days and different times (85.44%), but only 57.33% of those answers were correct (Suárez et al. 2024). These findings indicate that repeated consistency in model outputs does not necessarily ensure the correctness of the generated information. Yes–no questions generally showed high consistency across models. However, the fact that accuracy did not reach 100% underscores the need for caution when applying LLMs in this domain and the importance of verifying outputs with expert input. Furthermore, the observation that consistency was higher for yes–no questions but lower for open‐ended questions is also in line with previous reports (Gurbuz et al. 2024).

In our study, the differences observed in both content and consistency between the web‐based and mobile‐based interfaces of the same model were particularly noteworthy. Given the cloud‐based architecture of LLMs, their outputs would generally be expected to demonstrate similar levels of accuracy and consistency across interfaces. However, there are currently no peer‐reviewed studies directly comparing the performance of the same LLM across different interfaces. Existing assumptions regarding interface‐related differences are largely based on user experience rather than systematic scientific evaluation. In our findings, yes–no questions showed no significant differences in accuracy between web‐based and mobile‐based interfaces across all models. For open‐ended questions, however, ChatGPT‐4o showed comparatively higher GQS and mDISCERN scores on the web‐based interface, whereas Qwen2.5‐Max showed relatively higher scores on the mobile‐based interface. DeepSeek‐V3 and Gemini 2.0 Flash showed no significant differences between interfaces. Taken together, these findings suggest that interface‐related differences may influence the quality and consistency of LLM‐generated responses in TDI‐related scenarios, although the magnitude and underlying causes of these differences remain uncertain. Within the limitations of the present study, both model type and interface appear to be potential factors affecting response characteristics.

The observed differences in response quality and consistency among models and interfaces may be related to variations in model architecture and response‐generation processes. Potential contributing factors may include differences in training data, fine‐tuning strategies, and response‐generation settings. However, due to the closed‐source and cloud‐based nature of these systems, the underlying mechanisms responsible for these differences cannot be directly verified by users or independent researchers. Therefore, interpretations regarding the causes of performance differences across models or interfaces should be considered exploratory and interpreted with caution.

One of the key strengths of our study is that LLMs were evaluated using synchronized and standardized questions across both web‐based and mobile‐based interfaces. This approach enabled an objective comparison of interface‐related differences. In addition, the use of a question set based on the IADT 2020 guidelines and the high ICC values achieved between independent evaluators enhanced methodological reliability. Furthermore, evaluating responses using not only accuracy rates but also qualitative measures such as GQS and mDISCERN allowed for a more comprehensive assessment of response quality and reliability. Finally, the inclusion of temporal consistency analyses may contribute to the existing literature, as this aspect has been explored in relatively few previous studies.

Nevertheless, this study has several limitations. The question set was limited to 20 items and, although developed based on the 2020 IADT guidelines and reviewed by multiple endodontists to ensure clinical relevance and content validity, it does not capture the full spectrum of trauma scenarios encountered in practice. Future research may benefit from expanding the question pool and employing power analysis–based sampling to further strengthen methodological rigor. Additionally, only four LLMs were evaluated, and the findings may therefore not be generalizable to all available models. Moreover, the study did not include multimodal inputs such as radiographs or clinical photographs, which may play an important role in real‐world diagnostic decision‐making in TDI management. Although conversation history was cleared after each session and question order was standardized, minor prompt‐order or platform‐related response variations inherent to LLM systems cannot be completely excluded. Finally, the temporal evaluation period covered five consecutive days; while consistent with prior LLM stability research, longer‐term longitudinal follow‐up may provide a more comprehensive understanding of how model behavior evolves over time, particularly given the dynamic and continuously updated nature of LLMs.

## 5 Conclusion

In the present study, no significant differences were observed among models in yes–no questions. However, for open‐ended questions, Qwen2.5‐Max demonstrated comparatively higher GQS and mDISCERN scores, as well as relatively higher temporal consistency, than the other evaluated models. Moreover, the findings suggest that not only the choice of model, but also the interface through which the model is accessed (web‐based vs. mobile), can influence the quality and consistency of LLM‐generated responses. This indicates that the same model may behave differently depending on the platform through which it is used. Importantly, responses may not always be completely accurate, regardless of question type or interface. Therefore, information obtained from LLMs should always be critically evaluated and verified by qualified dental professionals, rather than being used as a sole basis for clinical decision‐making. Given the observational design and limited scope of the present study, further large‐scale and multimodal investigations are warranted to better understand the clinical reliability and applicability of LLMs in TDI‐related scenarios.

## Author Contributions

Ezgi Can Cekic designed the study. Ezgi Can Cekic, Mertkan Kumru, Burcu Yilmaz, Oğuz Tavsan, and Berk Celikkol performed the statistical analysis. Ezgi Can Cekic, Mertkan Kumru, and Burcu Yilmaz wrote the main manuscript text. Ezgi Can Cekic, Oğuz Tavsan, and Berk Celikkol prepared the tables. Ezgi Can Cekic and Oğuz Tavsan prepared the figures. All authors reviewed the manuscript.

## Funding

The authors have nothing to report.

## Ethics Statement

This study did not involve any clinical interventions, human or animal participants, or the use of identifiable patient data; therefore, formal ethical approval was not required.

## Conflicts of Interest

The authors declare no conflicts of interest.

## Data Availability

The data underlying this article will be shared on reasonable request to the corresponding author.

## References

[cre270416-bib-0001] Anagnostidis, S. , and J. Bulian . “How Susceptible Are LLMs to Influence in Prompts?” arXiv preprint arXiv:240811865 2024. 10.48550/arXiv.2408.11865.

[cre270416-bib-0002] Atil, B. , S. Aykent , A. Chittams , et al., “Non‐Determinism of “Deterministic” LLM Settings”. arXiv preprint arXiv:240804667 2024. 10.48550/arXiv.2408.04667.

[cre270416-bib-0003] Ayorinde, A. , D. O. Mensah , J. Walsh , et al. 2024. “Health Care Professionals' Experience of Using AI: Systematic Review With Narrative Synthesis.” Journal of Medical Internet Research 26: e55766. 10.2196/55766. 39476382 PMC11561443

[cre270416-bib-0004] Bourguignon, C. , N. Cohenca , E. Lauridsen , et al. 2020. “International Association of Dental Traumatology Guidelines for the Management of Traumatic Dental Injuries: 1. Fractures and Luxations.” Dental Traumatology 36, no. 4: 314–330. 10.1111/edt.12578. 32475015

[cre270416-bib-0005] Çege, E. E. , H. Cömert , N. Akal , and A. Ölmez . 2025. “Evaluation of the Performance of Artificial Intelligence Based Chatbots in Providing First Aid Information on Dental Trauma According to the ToothSOS Application.” Dental Traumatology 41: 696–705. 10.1111/edt.13078. 40488605 PMC12605821

[cre270416-bib-0006] Day, P. F. , M. T. Flores , A. C. O'Connell , et al. 2020. “International Association of Dental Traumatology Guidelines for the Management of Traumatic Dental Injuries: 3. Injuries in the Primary Dentition.” Dental Traumatology 36, no. 4: 343–359. 10.1111/edt.12576. 32458553

[cre270416-bib-0007] Fouad, A. F. , P. V. Abbott , G. Tsilingaridis , et al. 2020. “International Association of Dental Traumatology Guidelines for the Management of Traumatic Dental Injuries: 2. Avulsion of Permanent Teeth.” Dental Traumatology 36, no. 4: 331–342. 10.1111/edt.12573. 32460393

[cre270416-bib-0008] Gokkurt Yilmaz, B. N. , F. Ozbey , and B. E. Yilmaz . 2025. “Evaluation of the Performance of Different Large Language Models on Head and Neck Anatomy Questions in the Dentistry Specialization Exam in Turkey.” Surgical and Radiologic Anatomy 47, no. 1: 211. 10.1186/s12903-025-05926-2. 40983800

[cre270416-bib-0009] Guan, B. , T. Roosta , P. Passban , and M. Rezagholizadeh . “The Order Effect: Investigating Prompt Sensitivity to Input Order in LLMs.” arXiv preprint arXiv:250204134 2025. 10.48550/arXiv.2502.04134.

[cre270416-bib-0010] Gurbuz, T. , O. Gokmen , B. Devranoglu , A. Yurci , and A. A. Madenli . 2024. “Artificial Intelligence in Reproductive Endocrinology: An In‐Depth Longitudinal Analysis of ChatGPTv4's Month‐by‐Month Interpretation and Adherence to Clinical Guidelines for Diminished Ovarian Reserve.” Endocrine 86, no. 3: 1171–1177. 10.1007/s12020-024-04031-8. 39341951

[cre270416-bib-0011] Guven, Y. , O. T. Ozdemir , and M. Y. Kavan . 2025. “Performance of Artificial Intelligence Chatbots in Responding to Patient Queries Related to Traumatic Dental Injuries: A Comparative Study.” Dental Traumatology 41, no. 3: 338–347. 10.1111/edt.13020. 39578674

[cre270416-bib-0012] Hackl, V. , A. E. Müller , M. Granitzer , and M. Sailer . 2023. “Is GPT‐4 a Reliable Rater? Evaluating Consistency in GPT‐4's Text Ratings.” Frontiers in Education 8: 1272229. 10.3389/feduc.2023.1272229.

[cre270416-bib-0013] Johnson, A. J. , T. K. Singh , A. Gupta , et al. 2025. “Evaluation of Validity and Reliability of AI Chatbots as Public Sources of Information on Dental Trauma.” Dental Traumatology 41, no. 2: 187–193. 10.1111/edt.13000. 39417352

[cre270416-bib-0014] Khan, M. K. , and M. K. Jindal . 2023. “Random Tree Algorithm to Analyse the Relation Between Type of Traumatic Dental Injuries and Its Demographic and Predisposing Factors‐A Cross‐Sectional Study.” Indian Journal of Dental Research 34, no. 2: 114–118. 10.4103/ijdr.ijdr_846_21. 37787195

[cre270416-bib-0015] Kilinc, D. D. , and G. Sayar . 2019. “Assessment of Reliability of YouTube Videos on Orthodontics.” Turkish Journal of Orthodontics 32, no. 3: 145–150. 10.5152/TurkJOrthod.2019.18064. 31565689 PMC6756568

[cre270416-bib-0016] Künzle, P. , and S. Paris . 2024. “Performance of Large Language Artificial Intelligence Models on Solving Restorative Dentistry and Endodontics Student Assessments.” Clinical Oral Investigations 28, no. 11: 575. 10.1007/s00784-024-05968-w. 39373739 PMC11458639

[cre270416-bib-0017] Kuru, H. E. , A. Aşık , and D. M. Demir . 2025. “Can Artificial Intelligence Language Models Effectively Address Dental Trauma Questions?” Dental Traumatology 41: 567–580. 10.1111/edt.13063. 40170270 PMC12424120

[cre270416-bib-0031] Landis, J. R. , and G. G. Koch . 1977. “The Measurement of Observer Agreement for Categorical Data.” Biometrics 33, no. 1: 159–174. 10.2307/2529310. 843571

[cre270416-bib-0018] Levin, L. , P. F. Day , L. Hicks , et al. 2020. “International Association of Dental Traumatology Guidelines for the Management of Traumatic Dental Injuries: General Introduction.” Dental Traumatology 36, no. 4: 309–313. 10.1111/edt.12574. 32472740

[cre270416-bib-0019] Mustuloğlu, Ş. , and B. P. Deniz . 2025. “Evaluation of Chatbots in the Emergency Management of Avulsion Injuries.” Dental Traumatology 41: 437–444. 10.1111/edt.13041. 39865377 PMC12260122

[cre270416-bib-0020] Petti, S. , U. Glendor , and L. Andersson . 2018. “World Traumatic Dental Injury Prevalence and Incidence, a Meta‐Analysis—One Billion Living People Have Had Traumatic Dental Injuries.” Dental Traumatology 34, no. 2: 71–86. 10.1111/edt.12389. 29455471

[cre270416-bib-0021] Qiang, S. , H. Zhang , Y. Liao , et al. 2025. “Application of Large Language Models in Stroke Rehabilitation Health Education: 2‐Phase Study.” Journal of Medical Internet Research 27: e73226. 10.2196/73226. 40694436 PMC12306586

[cre270416-bib-0022] Rasool, Z. , S. Kurniawan , S. Balugo , et al. 2024. “Evaluating LLMs on Document‐Based QA: Exact Answer Selection and Numerical Extraction Using Cogtale Dataset.” Natural Language Processing Journal 8: 100083. 10.1016/j.nlp.2024.100083.

[cre270416-bib-0023] Sallam, M. , I. M. Alasfoor , S. W. Khalid , et al. 2025. “Chinese Generative AI Models (DeepSeek and Qwen) Rival ChatGPT‐4 in Ophthalmology Queries With Excellent Performance in Arabic and English.” Narra Journal 5, no. 1: e2371. 10.52225/narra.v5i1.2371. PMC12059827 40352182

[cre270416-bib-0024] Suárez, A. , V. Díaz‐Flores García , J. Algar , M. Gómez Sánchez , M. Llorente de Pedro , and Y. Freire . 2024. “Unveiling the ChatGPT Phenomenon: Evaluating the Consistency and Accuracy of Endodontic Question Answers.” International Endodontic Journal 57, no. 1: 108–113. 10.1111/iej.13985. 37814369

[cre270416-bib-0025] Tang, J. , J. Lai , J. Bui , et al. 2023. “Impact of Different Artificial Intelligence User Interfaces on Lung Nodule and Mass Detection on Chest Radiographs.” Radiology: Artificial intelligence 5, no. 3: e220079. 10.1148/ryai.220079. 37293345 PMC10245182

[cre270416-bib-0026] Yilmaz, B. E. , B. N. Gokkurt Yilmaz , and F. Ozbey . 2025. “Artificial Intelligence Performance in Answering Multiple‐Choice Oral Pathology Questions: A Comparative Analysis.” BMC Oral Health 25, no. 1: 573. 10.1007/s00276-025-03723-8. 40234873 PMC11998383

[cre270416-bib-0027] Zhou, L. , W. Schellaert , F. Martínez‐Plumed , Y. Moros‐Daval , C. Ferri , and J. Hernández‐Orallo . 2024. “Larger and More Instructable Language Models Become Less Reliable.” Nature 634, no. 8032: 61–68. 10.1038/s41586-024-07930-y. 39322679 PMC11446866

[cre270416-bib-0028] Zhu, G. , X. Zhang , and C. Chen . 2025. “Assessing and Enhancing the Reliability of Chinese Large Language Models in Dental Implantology.” BMC Oral Health 25, no. 1: 1242. 10.1186/s12903-025-06648-1. 40713564 PMC12296590

[cre270416-bib-0029] Zhu, S. , W. Hu , Z. Yang , J. Yan , and F. Zhang . 2025. “Qwen‐2.5 Outperforms Other Large Language Models in the Chinese National Nursing Licensing Examination: Retrospective Cross‐Sectional Comparative Study.” JMIR Medical Informatics 13: e63731. 10.2196/63731. 39793017 PMC11759905

[cre270416-bib-0030] Zieglmeier, V. , and A. M. Lehene . “Designing Trustworthy User Interfaces.” In: *Proceedings of the 33rd Australian Conference on Human‐Computer Interaction*, 2021: 182–189.

